# The Contribution of the Left Phrenic Nerve to Innervation of the Esophagogastric Junction

**DOI:** 10.1002/ca.23502

**Published:** 2019-12-02

**Authors:** Kati Haenssgen, Gudrun Herrmann, Annette Draeger, Manfred Essig, Valentin Djonov

**Affiliations:** ^1^ Institute of Anatomy University of Bern Bern Switzerland; ^2^ Department Gastroenterology, Tiefenau Hospital University of Bern Bern Switzerland

**Keywords:** esophagogastric junction, left phrenic nerve, phrenic plexus, celiac ganglion, celiac plexus, left phrenic inferior artery, caudal phrenic artery, humans, piglets, microdissection, nerve staining

## Abstract

The contribution of the left phrenic nerve to innervation of the esophagogastric junction. The esophagogastric junction is part of the barrier preventing gastroesophageal reflux. We have investigated the contribution of the phrenic nerves to innervation of the esophagogastric junction in humans and piglets by dissecting 30 embalmed human specimens and 14 piglets. Samples were microdissected and nerves were stained and examined by light and electron microscopy. In 76.6% of the human specimens, the left phrenic nerve participated in the innervation of the esophagogastric junction by forming a neural network together with the celiac plexus (46.6%) or by sending off a distinct phrenic branch, which joined the anterior vagal trunk (20%). Distinct left phrenic branches were always accompanied by small branches of the left inferior phrenic artery. In 10% there were indirect connections with a distinct phrenic nerve branch joining the celiac ganglion, from which celiac plexus branches to the esophagogastric junction emerged. Morphological examination of phrenic branches revealed strong similarities to autonomic celiac plexus branches. There was no contribution of the left phrenic nerve or accompanying arteries from the caudal phrenic artery in any of the piglets. The right phrenic nerve made no contribution in any of the human or piglet samples. We conclude that the left phrenic nerve in humans contributes to the innervation of the esophagogastric junction by providing ancillary autonomic nerve fibers. Experimental studies of the innervation in pigs should consider that neither of the phrenic nerves was found to contribute. Clin. Anat. 33:265–274, 2020. © 2019 Wiley Periodicals, Inc.

**Abbreviations**
EGJ: esophagogastric junction
LES: lower esophageal sphincter
LIPA: left inferior phrenic artery
TH: tyrosine hydroxylase

## INTRODUCTION

The esophagogastric junction (EGJ) can be classified according to anatomical, histological, physiological, and endoscopic criteria (Huang, 2018). Anatomically, it is the region between the lower esophagus and the gastric cardia of the proximal stomach. Histologically and endoscopically it is the transition from the stratified squamous epithelium of the esophagus to the simple columnar epithelium of the stomach (Huang, 2018). The EGJ regulates the passage of food and is an important part of the antireflux barrier (Mittal and Balaban, 1997). Maintenance of the antireflux barrier is essential for preventing gastroesophageal reflux disease, which is common among humans with a prevalence of 20% in Western populations, in which up to one third develop reflux esophagitis (Antunes and Curtis, 2018). Functional maintenance of the antireflux barrier is secured by the internal smooth muscle of the functional lower esophageal sphincter (LES) and the clasp and sling muscle fibers of the proximal stomach. Also, the external sphincter consists of the crural diaphragm with its right crus forming the esophageal hiatus. The phrenicoesophageal ligament connects the LES to the diaphragm (Mittal and Balaban, 1997).

The neural coordination of the internal and external sphincters is not yet completely understood, in particular during transient lower esophageal sphincter relaxation, which is considered to be the primary mechanism underpinning gastroesophageal reflux (Young et al., 2010). The diversity of nerve fibers involved complicates the matter. The LES and proximal stomach receive their innervation from the enteric nervous plexus, which in turn is subject to regulation by the autonomic nervous system. The celiac ganglion and celiac plexus provide sympathetic postganglionic, parasympathetic preganglionic, and afferent nerve fibers to the upper abdominal organs (Sişu et al., 2012). These fibers form a neural plexus that surrounds homonymous arteries such as the left gastric, hepatic, and inferior phrenic arteries. The individual contributions of the three plexuses to the pertinent innervation of the EGJ vary equally, both in extent and with respect to the connections to other plexuses and to vagal branches emanating from the anterior vagal trunk (Mitchell, 1940). The anterior and posterior vagal trunks provide the parasympathetic and afferent innervation, the anterior trunk dividing into gastric and hepatic branches and the posterior trunk into gastric and celiac branches.

The crural diaphragm is a striated muscle innervated by the left and right phrenic nerves, both of which run through the thoracic cavity before penetrating the diaphragm (Muller Botha, 1957). Their posterior branches innervate the crural diaphragm and, accordingly, the esophageal hiatus, and then continue as phrenicoabdominal branches within the abdominal cavity, where they innervate visceral and peritoneal structures (Kostreva and Pontus, 1993). Fine extensions of them can connect to branches of the celiac plexus, commonly known as the phrenic plexus (subsequently referred to as celiac plexus branches). The junction sometimes contains a sympathetic phrenic ganglion (Tubbs et al., 2008). This is more common on the right, but also occurs on the left side with variable frequency (Loukas et al., 2016). The phrenic plexuses innervate the upper parts of the stomach, more constantly from the left than the right side (Mitchell, 1940). No direct branches from the right phrenic nerve to the EGJ have been described. In contrast, participation of the left phrenic nerve in the innervation of the EGJ is attested in textbooks (Braus and Elze, 1960) and corroborated experimentally: left phrenicotomy in dogs resulted in more rapid gastric emptying with accumulation of gas in the fundus and gastric hypotonia and hypokinesia (Jefferson and Necheless, 1948). Morphological investigations identified a “gastric branch of the left phrenic nerve” (Mitchell, 1940), but this has not been systematically examined in a bigger group of specimens.

Esophagogastric anatomy and physiology is similar in porcine and human stomachs (Vicente et al., 2001). Pigs are also prone to spontaneous reflux (Kadirkamanathan et al., 1999) and are frequently used for testing surgical procedures. Despite the anatomical similarity there are species‐specific differences mainly related to the transition of the squamocolumnar junction and the thickness of the musculature at the EGJ (Vicente et al., 2001), and also to the arterial supply of the stomach and the diaphragm. While the crural diaphragm in humans receives its blood supply from the right and left inferior phrenic arteries, there is a single artery in pigs, the caudal phrenic artery.

Comprehensive knowledge on the innervation pattern of the EGJ is important for understanding the mechanisms that underlie reflux. The aim of this study was to investigate the abdominal phrenic nerves and their contribution to the innervation of the EGJ in humans systematically. The frequency, the course, the endings, and connections of left phrenic nerve branches were examined and compared to those in piglets in order to evaluate this animal model.

## MATERIALS AND METHODS

The 30 adult human specimens used in this study (14 males and 16 females aged 65–96 years, with a mean of 84.4 years) comprised 22 embalmed according to Thiel (Thiel, 1992) and eight embalmed according to a conventional protocol for student courses. They were all obtained from the body donation program of the Institute of Anatomy, University of Bern. The human material was used in accordance with the Guidelines of the Swiss Academy of Medical Sciences (Rütsche and D'Amico, 2015). Fourteen piglets were obtained from the Swine clinic at the Vetsuisse Faculty, University of Bern. The piglets were either stillborn or found dead within 1–3 days after birth.

### Macroscopic Dissection (Humans)

The thoracic cavity was opened in all bodies and the heart and lungs were removed, while the pericardium was preserved along with the right and left phrenic nerves, the right and left vagal nerves, the esophagus, the aorta, and the sympathetic trunks. The abdominal cavity was opened and the diaphragmatic attachments to the ribcage were dissected so the esophageal hiatus could be accessed. The upper abdominal organs remained in their original topographical positions. All the structures between the crura of the lumbar diaphragm and their peritoneal sheath were completely dissected in all bodies. The left phrenic nerve was dissected from the point where it pierces the diaphragm to its terminal branches. In addition, the left inferior phrenic artery (LIPA) and the left‐sided celiac ganglion were carefully dissected. The abdominal esophagus and upper stomach with the anterior and the posterior vagal trunk and the branches of the left gastric artery were carefully dissected in all bodies. The left phrenic nerve and in particular its posterior branch was identified in each specimen. The posterior branch consistently partitioned into three main branches: lateral, middle, and medial. The course of each branch was mapped using a binocular microscope.

### Histological Processing of Nerve Samples (Humans)

Nerve samples were embedded in paraffin and the sections (8 μm) were stained with hematoxylin/eosin or Goldner. Samples were also prepared for electron microscopy. They were taken from the distal ends of the:

- left phrenic nerve branches before connecting to celiac plexus branches
- celiac plexus branches before connecting to left phrenic nerve branches

### Macroscopic Dissection (Piglets)

Four piglets (500–1,200 g body weight) were preserved in 4% formaldehyde and dissected macroscopically. The preparatory steps corresponded to those for human macrodissection.

### Nerve Staining (Piglets)

Sihler's whole mount nerve staining technique was applied to 10 piglets (Mu and Sanders, 2010). In four of them, the diaphragm was detached from its bony frame and isolated with esophagus and stomach attached. In the other six, an arterial cast was prepared using colored silicone (Haenssgen et al., 2014) before the staining protocol was applied in order to visualize the topographic relationship between arteries and nerves within the muscular tissue. In these piglets, the attachment of the crural diaphragm to the lumbar part of the spine remained intact.

All histological samples were examined and documented using a Zeiss Axio Imager M2 microscope. Images were captured with a Canon EOS 5 D Camera (Canon EOS 5D, Japan) and processed with Photo Paint and Corel Draw Graphics Suite 2018 (Corel Corporation). Microdissection was undertaken using a dissection microscope (OPMI® Pico, Carl Zeiss Surgical GmbH, Germany).

## RESULTS

### Macroscopic Dissection of Human Specimens

In 23 of the 30 specimens (76.6%), the left phrenic nerve was involved in the innervation of the EGJ. Three main patterns were identified and classified as variants A to C according to their frequency.

**Figure 1:**
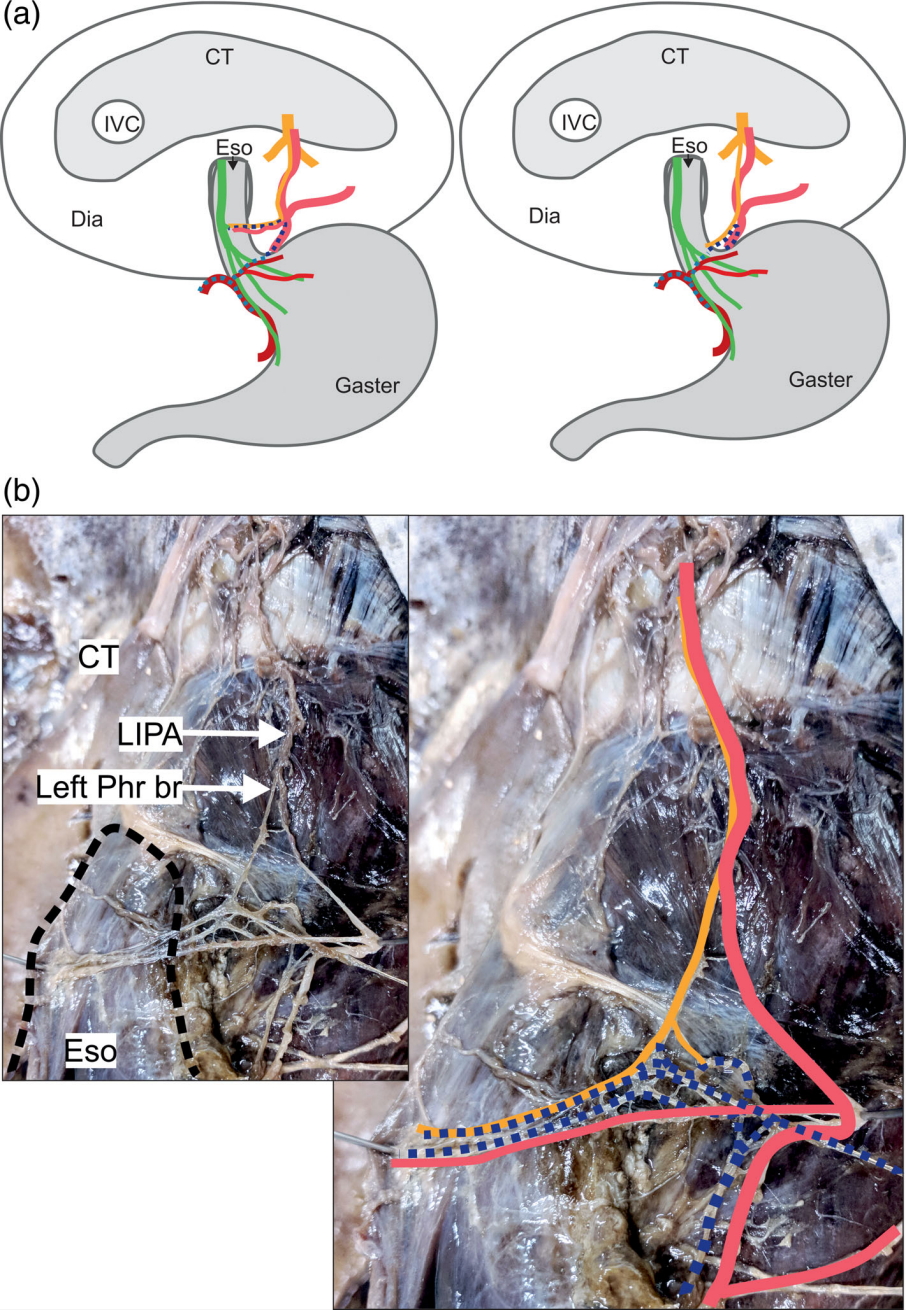
A delicate branch of the left phrenic nerve connects to celiac plexus branches (Variant A, *n* = 14). (**a**) Two examples for specimens of variant A reveal innervation of the abdominal esophagus by a delicate left phrenic nerve branch (yellow) connected to celiac plexus branches (dark blue dotted) together with an accompanying artery (left picture) of the left inferior phrenic artery (light red), and innervation of the esophagogastric junction without accompanying arteries (right picture) and connections to the left gastric plexus (light blue dotted). (**b**) The esophagus (black dotted line) lies within the esophageal hiatus (left picture). Several celiac plexus branches (dark blue dotted) connect to a delicate phrenic nerve branch (yellow). Together, they run to the abdominal esophagus accompanied by an arterial branch (light red). The anatomical structures are highlighted in color (right picture). CT = central tendon; Dia = diaphragm; Eso = esophagus, IVC = inferior caval vein; LIPA = left inferior phrenic artery; Phr br = phrenic nerve branch. Color code: green: anterior vagal trunk with gastric branches; yellow: phrenic nerve with muscular branches and esophageal/esophagogastric branches; dark blue dotted: left celiac plexus branch; light blue dotted: left gastric plexus; dark red: left gastric artery; light red: left inferior phrenic artery. [Color figure can be viewed at http://wileyonlinelibrary.com]

*Variant A* was the most common and was found in 14 of the 30 specimens (46.6%). A very delicate left phrenic branch communicated with one to three celiac plexus branches that derived from the left side of the celiac ganglion (Fig. 1). Together, they formed a network that either reached the lower esophagus (measured from 2 cm above the angle of His up to the border of the esophageal hiatus) in seven specimens, or the EGJ (± 2 cm from the angle of His) in the other seven. At the EGJ, the terminal branches of this nerve plexus communicated with a branch originating from the anterior vagal trunk and the left gastric plexus. The LIPA provided an esophageal or esophagogastric branch to the EGJ in six specimens. The phrenic branch closely followed the LIPA in all but three specimens. In two of these, the nerve ran within the muscle of the left crural diaphragm before emerging and connecting to celiac plexus branches. In the other, it ran at a distance of 1 cm laterally to the LIPA before connecting to the celiac plexus branches. Nerves innervating the left suprarenal gland disengaged from the connection side between the phrenic and celiac plexus branches.

*Variant B* was found in six of the 30 specimens (20%) and was characterized by a thicker, distinct phrenic branch following different courses distinguished as subgroups (below). In some but not all cases, these phrenic branches connected with celiac plexus branches. In others they connected with branches of the left gastric and/or hepatic plexus on the right side of the EGJ. Another characteristic was the direct connection with vagal branches of the anterior vagal trunk and accompanying arteries of the LIPA in all specimens.

**Figure 2:**
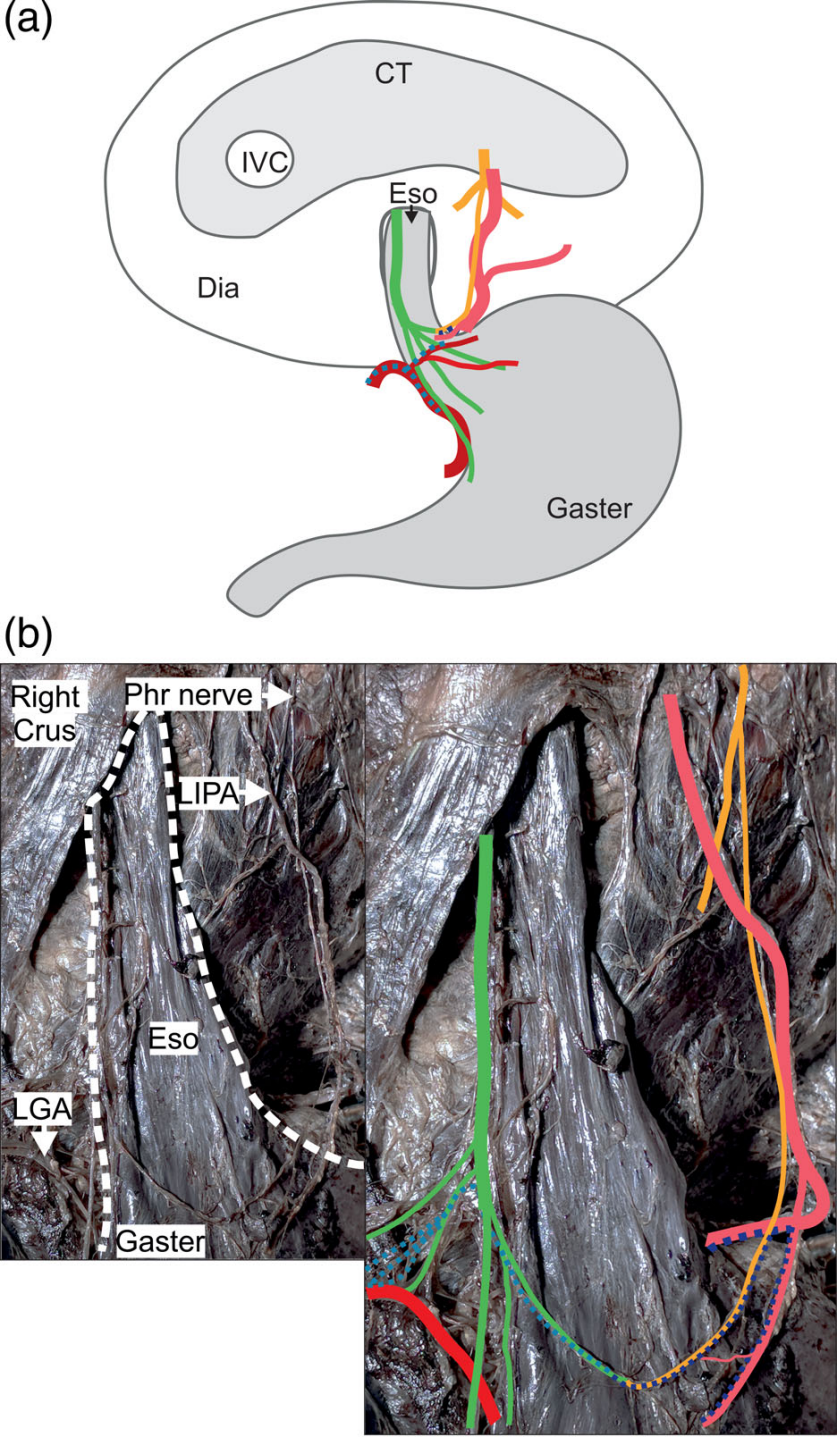
A distinct branch of the left phrenic nerve connects to a vagal branch at the esophagogastric junction (Variant B, *n* = 6). (**a**) One example of a variant B specimen. A distinct phrenic nerve branch (yellow) connects to celiac plexus branches (dark blue dotted) and runs to the esophagogastric region, where it connects to a branch of the anterior vagal trunk (green). All the specimens showed esophageal/esophagogastric arteries of the left inferior phrenic artery (light red). (**b**) The pictures show one specimen of subgroup B1 (*n* = 3). The white dotted line surrounds the esophagus and the proximal part of the gaster (left picture). The phrenic nerve branch (yellow) connects to a gastric branch of the anterior vagal trunk (green) and to small celiac plexus branches (dark blue dotted) and the left gastric plexus (light blue dotted). The anatomical structures are highlighted in color (right picture). CT = central tendon; Dia = diaphragm; Eso = esophagus, IVC = inferior caval vein; LGA = left gastric artery; LIPA = left inferior phrenic artery; Phr = phrenic nerve (posterior part). Color code: green: anterior vagal trunk with gastric branches, yellow: phrenic nerve with muscular branches and esophageal/esophagogastric branch; dark blue dotted: left celiac‐nerve branches; light blue dotted: left gastric plexus; dark red: left gastric artery; light red: left inferior phrenic artery. [Color figure can be viewed at http://wileyonlinelibrary.com]

**Figure 3:**
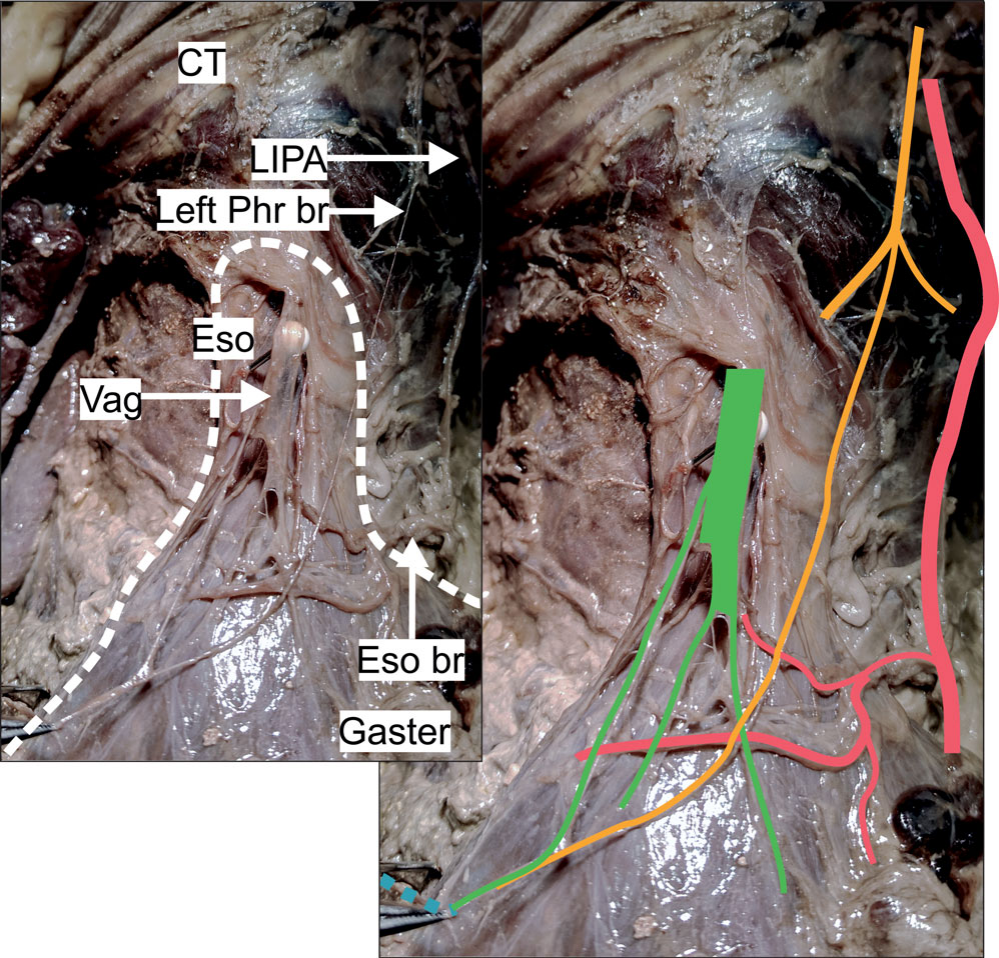
The pictures show one specimen of subgroup B2 (*n* = 2). The white dotted line surrounds the esophagus and proximal part of the gaster (left picture). The phrenic nerve branch (yellow) follows a steep course before connecting to a gastric branch of the anterior vagal trunk (green). It connected not to celiac plexus branches from the left side but to branches of the left gastric plexus (light blue dotted). The anatomical structures are highlighted in color (right picture). CT = central tendon; Eso = esophagus; Eso br = arterial esophageal/esophagogastric branch; LIPA = left inferior phrenic artery; Phr br = phrenic nerve branch; Vag = anterior vagal trunk. Color code: green: anterior vagal trunk with gastric branches, yellow: phrenic nerve with muscular branches and esophageal/esophagogastric branch; light blue dotted: left gastric plexus; light red: left inferior phrenic artery. [Color figure can be viewed at http://wileyonlinelibrary.com]

**Figure 4:**
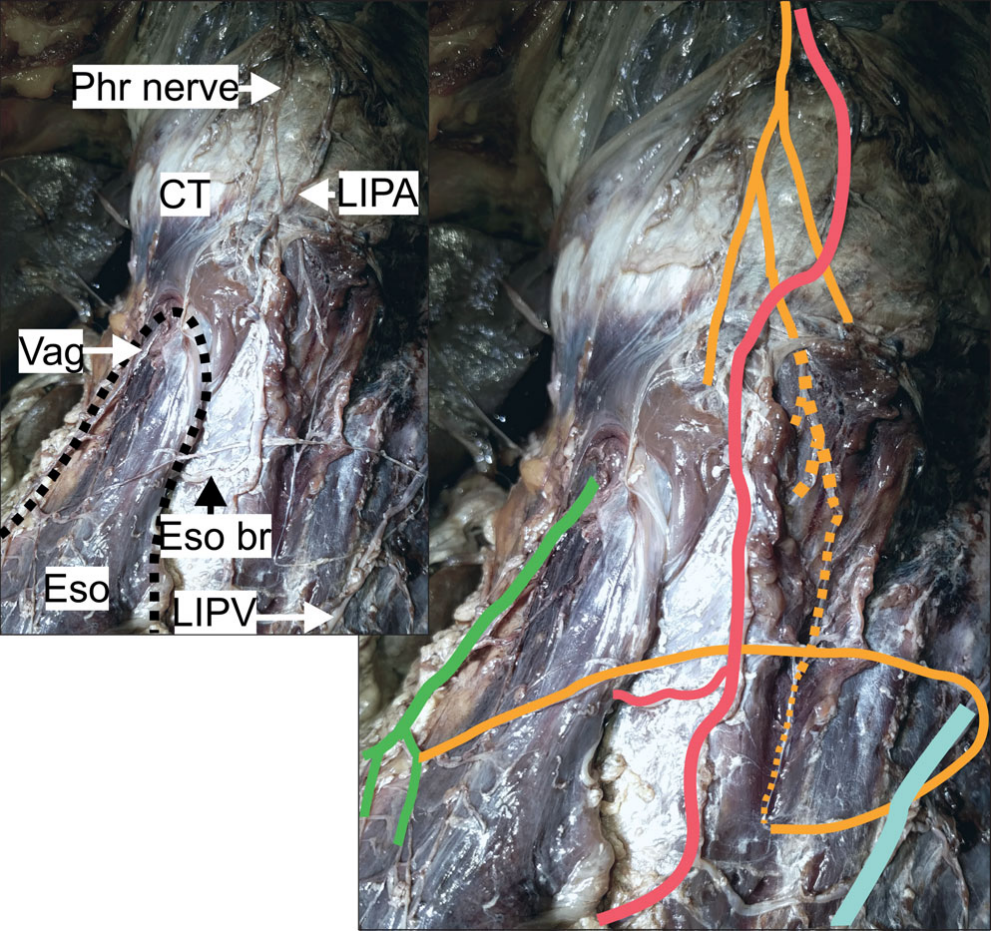
The pictures show the specimen of subgroup B3 (*n* = 1). The black dotted line surrounds the esophagus (left picture). The phrenic nerve branch (yellow) runs within the left crural diaphragm (yellow dotted line) before forming a loop back to the abdominal esophagus and connecting to a gastric branch of the anterior vagal trunk (green). The anatomical structures are highlighted in color (right picture). CT = central tendon; Eso = esophagus; Eso br = arterial esophageal/esophagogastric branch; LIPA = left inferior phrenic artery; LIPV = left inferior phrenic vein; Phr = phrenic nerve (posterior branch); Vag = anterior vagal trunk. Color code: green: anterior vagal trunk with gastric branches, yellow: phrenic nerve with muscular branches and esophageal/esophagogastric branches; light red: left inferior phrenic artery; light blue: left inferior phrenic vein. [Color figure can be viewed at http://wileyonlinelibrary.com]

*Subgroups of B*: The course of the phrenic branch had such clearly distinct variants that it was necessary to subclassify it (Figs. 2, 3, 4).

*Subgroup B1*. In three specimens, a clearly distinguishable phrenic branch accompanied the LIPA and connected to small celiac plexus branches before joining vagal branches at the EGJ (Fig. 2). Branches of the left gastric plexus in two of these specimens and of the hepatic plexus in the other ran alongside the vagal branches and completed the innervation.

*Subgroup B2*: In two specimens, the phrenic branch did not accompany the LIPA. Instead, it took a steep descending course, passing to the right side of the abdominal esophagus before communicating with a branch of the anterior vagal trunk and the left gastric plexus (Fig. 3).

*Subgroup B3*: In one specimen, the phrenic branch entered the muscular part of the left crural diaphragm and ran downward for approximately 5 cm, giving off muscular branches. It surfaced and turned sharply left to enter the anterior surface of the abdominal esophagus and communicated with a vagal branch (Fig. 4). There were no connections with other plexuses.

**Figure 5:**
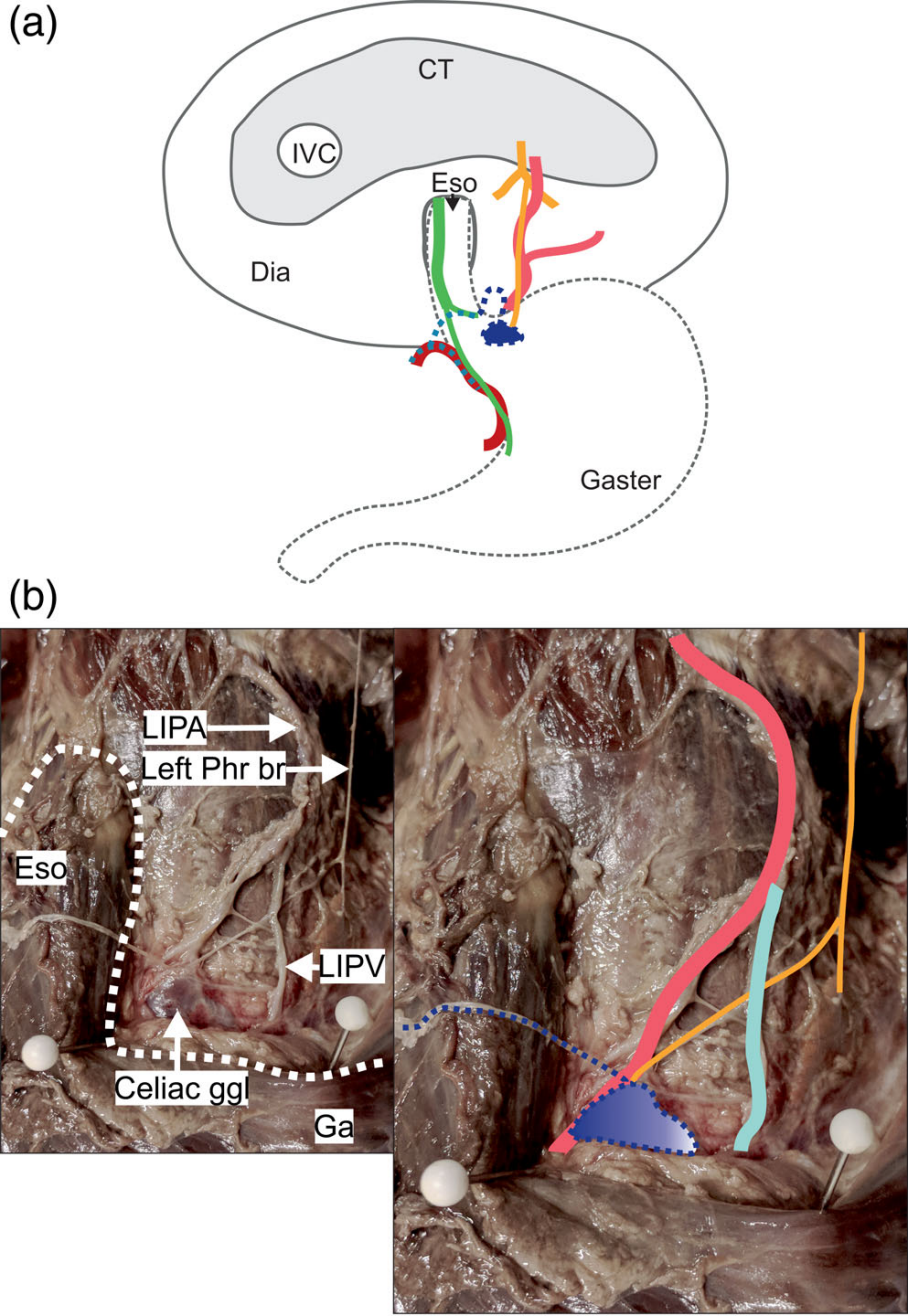
A distinct branch of the left phrenic nerve directly connects to the celiac ganglion (Variant C, *n* = 3). (**a**) The phrenic nerve branch (yellow) connects to the left‐sided celiac ganglion (dark blue). A celiac plexus branch (dark blue dotted) forms a wide loop in front of the esophagogastric junction and connects to other plexuses. No arterial branches of the left inferior artery (light red) accompany the nerve. (**b**) The white dotted line surrounds the esophagus and the proximal part of the gaster (left picture). The left phrenic branch (yellow) connects to the left‐sided celiac ganglion (dark blue). The anatomical structures are highlighted in color (right picture). CT = central tendon; Dia = diaphragm; Eso = esophagus, Ga = gaster; ggl = ganglion; IVC = inferior caval vein; LIPA = left inferior phrenic artery; LIPV = left inferior phrenic vein; Phr br = phrenic nerve branch. Color code: green: anterior vagal trunk with gastric branches, yellow: phrenic nerve with muscular branches and branch to the ganglion; dark blue dotted: left celiac‐nerve branches; light blue dotted: left gastric plexus; dark red: left gastric artery; light red: left inferior phrenic artery; light blue: left inferior phrenic vein. [Color figure can be viewed at http://wileyonlinelibrary.com]

*Variant C*: In three specimens (10%), the left phrenic branch contacted the left‐sided celiac ganglion directly (Fig. 5). A celiac plexus branch emerged from the ganglion and ran to the EGJ, thereby forming a wide loop before connecting to the anterior vagal trunk and the other plexus. No arteries accompanied the nerve.

Seven specimens (23.3%) had no phrenic branch or accompanying artery. The posterior vagal trunk did not connect directly to the left phrenic branch. On the other hand, the nerve fibers that branched off to the celiac ganglion, the celiac branches, were clearly identified.

### Light and Electron Microscopic Assessment of Human Nerve Samples

**Figure 6:**
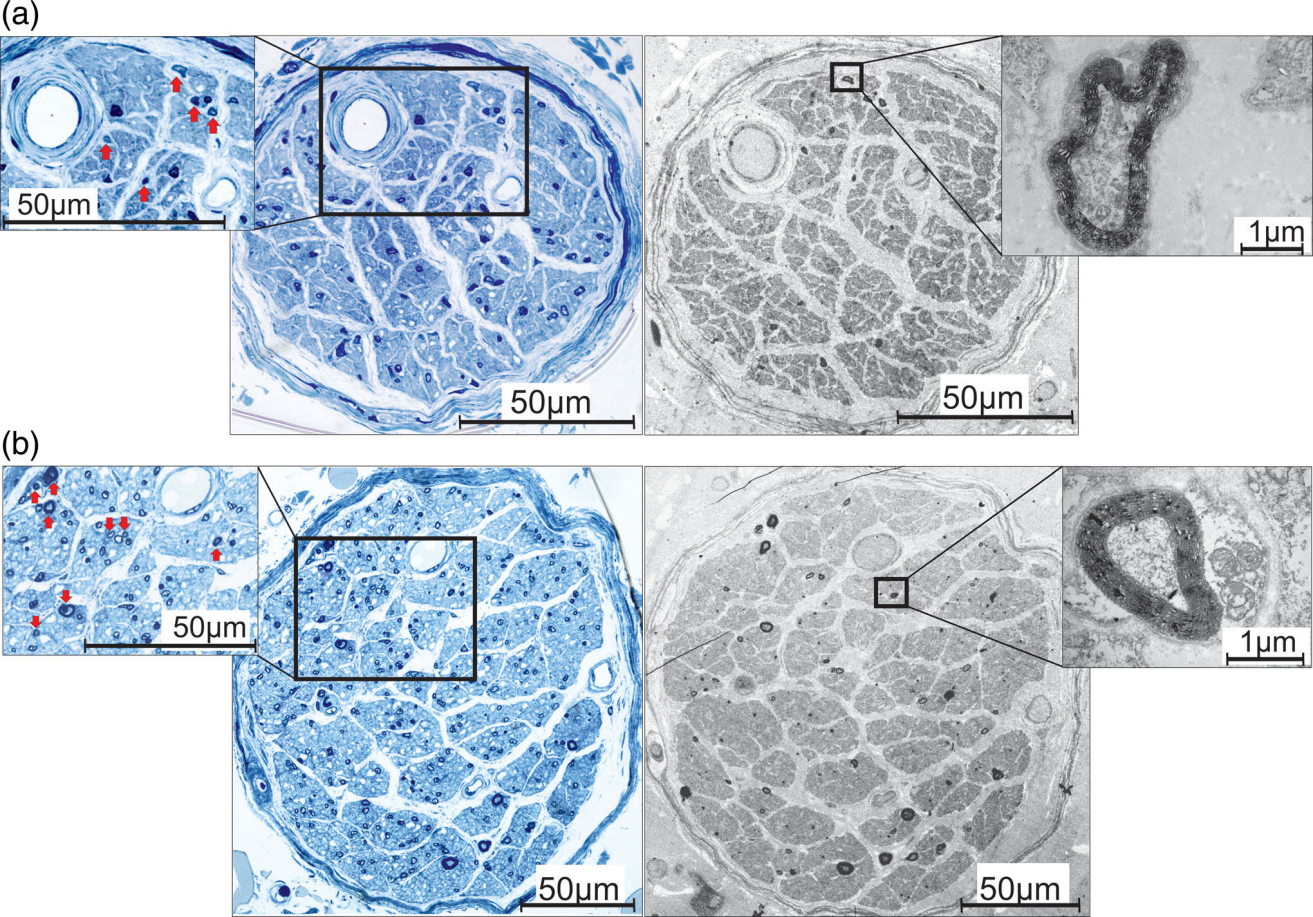
The nerve fiber compositions of (**a**) a human phrenic nerve branch and (**b**) a human celiac plexus branch. The semithin sections (left blue pictures) and the TEM sections (right pictures) show several distinct fascicles containing areas of nonmyelinated axons with interspersed small‐diameter myelinated axons. The zoom‐in (left pictures) demonstrates myelinated axons (arrows) at low magnification. The remaining parts of the nerves consist of nonmyelinated axons. The zoom‐in (right pictures) shows small‐diameter myelinated axons at high magnification. [Color figure can be viewed at http://wileyonlinelibrary.com]

Samples taken from the distal ends of the phrenic nerve and the celiac plexus branches revealed distinct fascicles dominated by areas of nonmyelinated axons and few interspersed small‐diameter myelinated axons. The latter were more numerous in the larger celiac plexus branches (Fig. 6).

### Macroscopic Dissection and Nerve Staining in Piglets

**Figure 7:**
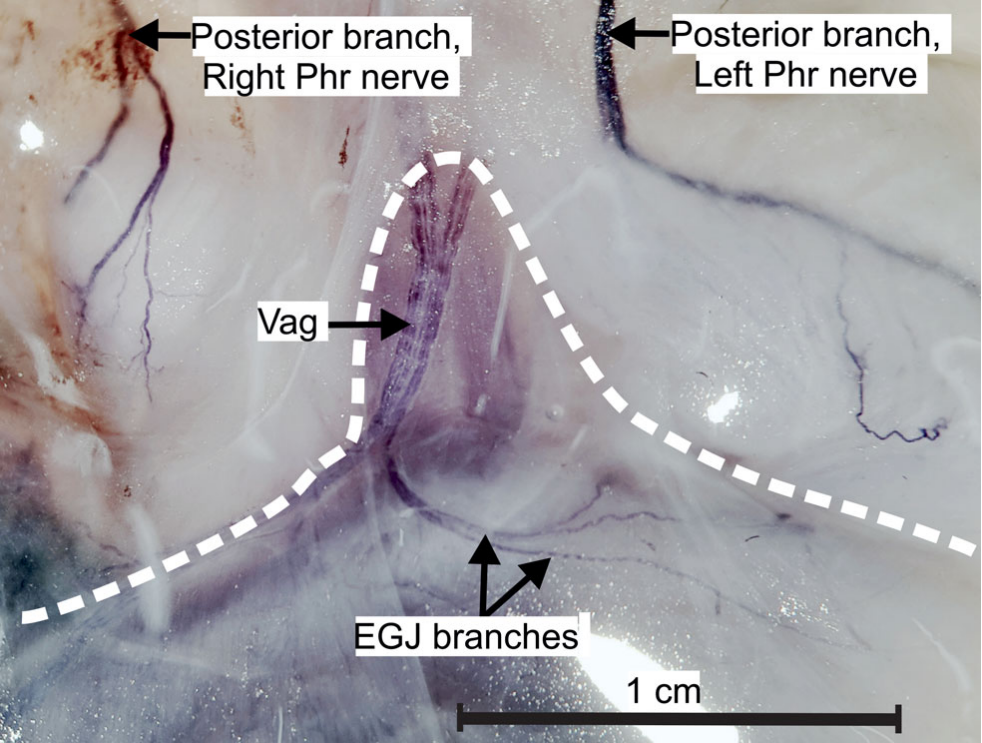
The nerves at the esophageal hiatus in piglets. Sihler staining shows the course of the posterior branches of the right and left phrenic nerve. No branch from the phrenic nerves reached the esophagogastric junction and no connections between the left phrenic nerve and the anterior vagal trunk were observed. The white dotted line surrounds the esophagus and the proximal stomach. EGJ branches = branches of the anterior vagal trunk to the esophagogastric junction; Phr = phrenic nerve, Vag = anterior vagal trunk. [Color figure can be viewed at http://wileyonlinelibrary.com]

**Figure 8:**
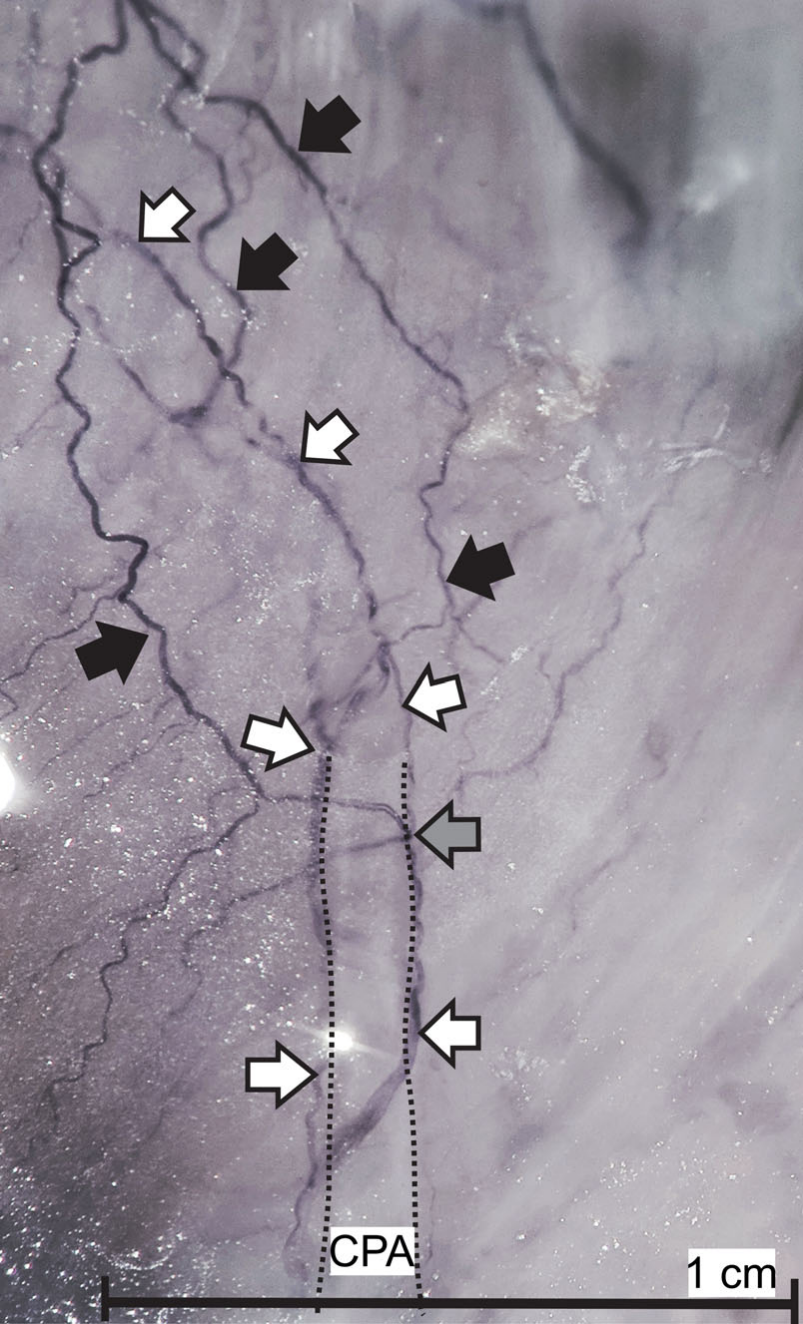
The main branch of the caudal phrenic artery within the musculature of the right crus in piglets. Sihler staining shows the accompanying celiac plexus branches entering the muscle. Celiac plexus branches (white arrows) run independently from phrenic nerve branches (black arrows). No connections between celiac plexus and phrenic branches were observed. The connection (gray arrow) is in reality a loop formed by the phrenic branch circling the celiac plexus branch. The black dotted line comprises the caudal phrenic artery. CPA = caudal phrenic artery. [Color figure can be viewed at http://wileyonlinelibrary.com]

It was found that none of the piglets had phrenic branches to the EGJ and therefore no connections to vagal branches (Fig. 7). The posterior branch of the left phrenic nerve ran along the border between the central tendon and the muscular crural diaphragm. Nerve branches entered the left side of the esophageal hiatus and the left crus. Staining revealed the three‐dimensional nervous network within the muscle with fine branches to the diaphragmatic fascial covering and to the phrenicoesophageal ligament. One or two celiac plexus branches accompanied the caudal phrenic artery into the muscle but without connections with phrenic branches (Fig. 8). The caudal phrenic artery in piglets followed a very short course of about 5 mm before disappearing within the muscle. It did not release branches to the stomach, esophagus, or EGJ in any piglet. In contrast to the findings in humans, celiac plexus branches from the celiac ganglion always ran with arteries and did not form individual loops to the EGJ.

## DISCUSSION

The components of the EGJ are innervated by both vagal trunks and autonomic fibers derived from the celiac plexus. We were able to confirm previous findings regarding interindividual differences and the relative contributions of the different plexuses to innervation of the EGJ in humans (Mitchell, 1940). We also found that the left phrenic nerve contributed to innervation of the EGJ in 23 out of 30 (76.6%) human specimens but in none of the piglets. Among the humans, 14 showed a delicate phrenic nerve branch connecting to celiac plexus branches, which ran to the abdominal esophagus or the region of the EGJ. In three specimens, the phrenic branch connected not to celiac plexus branches but directly to the celiac ganglion. In six specimens, the left phrenic nerve was clearly distinguishable and finally connected to branches of the anterior vagal trunk at the anterior surface of the EGJ. A direct vagal‐phrenic nerve loop at the EGJ has not previously been described in humans. A delicate branch of the left phrenic nerve that runs to the viscus near the cardiac orifice has previously been identified in one specimen (Mitchell, 1940). Our systematic study has revealed that these phrenic branches are highly variable in thickness, course, and interconnections with other autonomic plexuses and can even be absent, as in seven of the 30 specimens (23.3%). We demonstrated four different anatomical patterns through which the left phrenic nerve can contribute to innervation of the EGJ in humans:

1. a delicate left phrenic branch that connects to left‐sided celiac plexus branches;
2. a distinct left phrenic branch that connects to left‐sided celiac plexus branches;
3. a distinct left phrenic branch and separate left‐sided celiac plexus branches;
4. a distinct left phrenic branch contacting the left‐sided celiac ganglion and separate celiac plexus branches.

All these arrangements could reflect variants of the same basic structure: the left phrenic plexus, defined as the connection between phrenic nerve and celiac plexus branches (Müller, 1931). The main characteristics are the connections to other autonomic plexuses and communication with vagal branches. The correlation with esophageal or esophagogastric arterial branches of the LIPA is less obvious. These arteries were always present in specimens with a distinct phrenic branch connecting to vagal branches and were always absent in specimens with no identifiable phrenic branches. The variability in presentation of the left phrenic nerve branch and even its obvious absence in some specimens indicated that it is sympathetic, as also proposed by Mitchell (1940). It reflects the general pattern of variability of the plexuses that innervate the EGJ and it explains the direct connection to vagal branches. The sympathetic network is denser in sphincteric regions, including the LES (Furness et al., 2014), than in normal smooth muscle layers (Lomax et al., 2010).

The sympathetic nature of the phrenic branch is further supported by analysis of its fiber composition, which is similar to its counterpart in celiac plexus branches. Areas of nonmyelinated and small‐diameter myelinated fibers suggest the presence of autonomic and sensory nerves (Kandel et al., 2000). Sensory nerve endings in the phrenicoabdominal branches participate in innervation of the parietal peritoneum, in particular in the central region of the diaphragm (Struller et al., 2017). Afferent recordings from the right phrenic nerve in dogs have been associated with mechanosensitive areas in the hepatic veins, the liver, and the inferior vena cava (Kostreva and Pontus, 1993). Mechanosensory visceral afferents are small‐diameter myelinated fibers while painful sensations are transmitted via nonmyelinated fibers (Kandel et al., 2000). The phrenic nerve receives sympathetic fibers originating from the middle and lower cervical ganglia, from the sympathetic trunk, or from a sympathetic plexus on the pleural cupula (Yano, 1928). Recent studies have demonstrated tyrosine hydroxylase (TH)‐positive fibers in both phrenic nerves. This indicates sympathetic activity (Verlinden et al., 2018). These fibers were either homogeneously distributed or present in distinct areas or as separate fascicles within the nerve. Interestingly, there was no TH activity in the left phrenic nerve at the level of the diaphragm in four specimens investigated, which could be explained by sympathetic fibers leaving the nerve within the thoracic cavity (Verlinden et al., 2018). In this case, the phrenic nerve could regain sympathetic fibers from the branches of the celiac plexus, which use the phrenic nerve as a conduit, as proposed by Verlinden et al. (2018) for the right phrenic nerve in humans.

No convincing function has been proposed for sympathetic fibers in the phrenic nerve. An investigation of right phrenic nerves in cats suggests vasoconstrictor function within the diaphragmatic muscle (Bałkowiec and Szulczyk, 1992). The diversity and composition of the left phrenic nerve branch at the EGJ suggests ancillary functions in the innervation of blood vessels and/or internal sphincter muscles and for transmitting mechanosensitive and/or nociceptive stimuli. The latter could be important during events that lead to transient lower esophageal sphincter relaxation.

We could not demonstrate ancillary nerve fibers from either of the phrenic nerves in piglets. We do not think that the differences in age, body weight, and body size between the adult human specimens and the newborn piglets affected the assessment of the innervation. Human embryos of 25–30 mm length have ganglia of the sympathetic trunk, autonomic ganglia, and dense nervous networks surrounding the abdominal arteries, and nerves at the EGJ (authors’ unpublished data). We therefore assume that the general pattern of innervation already exists at birth and is not affected by body weight, size, or healthy aging, in contrast to the process of myelination, which progresses after birth. We can only speculate about the reasons why the innervation pattern at the EGJ in piglets is less variable and why the left phrenic nerve does not contribute to the innervation. Differences in embryonic development of the nerves innervating the EGJ could be a factor, especially as the development of autonomic nerves is probably interrelated with the development of the arteries supplying the EGJ and the diaphragm.

There is a critical difference between humans and pigs in the arterial supply to the EGJ. While the LIPA often provides arterial branches here, there were no such branches of the caudal phrenic artery in the piglets. The relationship between the innervation pattern and the arterial supply to the EGJ remains an interesting field for future studies. We presume that the demands on the mechanical properties of the antireflux barrier differ between tetrapods and humans. The species‐specific differences (Vicente et al., 2001) could provide pigs with sufficient protective mechanisms to render an additional nervous protection at the antireflux barrier obsolete. Nevertheless, the differences in innervation of the EGJ should be considered when results from studies of piglets are related to human studies that deal with mechanisms underlying gastroesophageal reflux.
